# Innovative solutions for disease management

**DOI:** 10.1186/s42234-023-00131-4

**Published:** 2023-12-06

**Authors:** Dafni Carmina, Valentina Benfenati, Claudia Simonelli, Alessia Rotolo, Paola Cardano, Nicoletta Grovale, Lorenza Mangoni di S. Stefano, Tiziana de Santo, Roberto Zamboni, Vincenzo Palermo, Michele Muccini, Francesco De Seta

**Affiliations:** 1 Medtronic Clinical & Regulatory Solutions – Study & Scientific Solutions, Via Aurelia 866, Roma, 00165 Italy; 2 Consiglio Nazionale delle Ricerche, Istituto per la Sintesi Organica e Fotoreattività, via Gobetti 101, Bologna, 40129 Italy; 3 Consiglio Nazionale delle Ricerche, Istituto per lo Studio dei Materiali Nanostrutturati, via Gobetti 101, Bologna, 40129 Italy; 4 Mister Smart Innovation S, via Gobetti 101, Bologna, 40129 Italy

**Keywords:** Disease management, Digital health, Patient-centric healthcare

## Abstract

The increasing prevalence of chronic diseases is a driver for emerging big data technologies for healthcare including digital platforms for data collection, systems for active patient engagement and education, therapy specific predictive models, optimized patient pathway models. Powerful bioelectronic medicine tools for data collection, analysis and visualization allow for joint processing of large volumes of heterogeneous data, which in turn can produce new insights about patient outcomes and alternative interpretations of clinical patterns that can lead to implementation of optimized clinical decisions and clinical patient pathway by healthcare professionals.

With this perspective, we identify innovative solutions for disease management and evaluate their impact on patients, payers and society, by analyzing their impact in terms of clinical outcomes (effectiveness, safety, and quality of life) and economic outcomes (cost-effectiveness, savings, and productivity).

As a result, we propose a new approach based on the main pillars of innovation in the disease management area, i.e. progressive patient care models, patient-centric approaches, bioelectronics for precise medicine, and lean management that, combined with an increase in appropriate private-public-citizen-partnership, leads towards Patient-Centric Healthcare.

## Introduction

Applying integrated care approaches in therapeutic areas with high prevalence, high resource consumption and unmet needs, can drive substantial improvement in outcomes for patients, caregivers, and society (Gabutti et al. 2017; Kruk et al. 2018; WHO, 2016; McPhail 2016). These models demand that patient needs are well understood and segmented, and that high-risk parts of the therapy specific patient pathway are identified. Approaches of this type become more important in the case of chronic patients, who experience a multi-year journey, often undermined by inconsistent, late and poor-quality care at typical points in the pathway (Gabutti et al. 2017). Best practice integrated care shares common hallmarks to overcome inefficiencies in patient management, such as patient-centric models (matching patients specific needs; proactive not reactive; home-centered and with education and coaching) and coordinated, interlinked teams (multi-disciplinary and connected teams; clearly assigned roles, responsibilities and communication) (Gabutti et al. 2017; Kruk et al. 2018; Di Somma et al. 2020).

At the global level, there is a significant heterogeneity of innovation practices, fragmented regulations for nascent markets, and new standards based on industry self-regulation (Garden et al. 2019). This generates uncertainty in relation to the regulatory framework. In addition, protocols and standard parameters of reference do not consider new scientific/technological knowledge, or the multiplicity of factors related to a condition, which may compromise the efficient implementation of more complex and disruptive innovation. The resources needed to finance new technologies is another challenge, considering the outcomes of some initiatives are associated to high uncertainty (Garden et al. 2019). Finally, the epicentrism of the health management systems, the centers of access to diagnosis, all cure and all long-term assistance, cause not only a minor therapeutic effect but also a persistent discomfort of patients and a worse quality of life of their caregiver (Dueñas et al. 2016).

Within this framework, integrated care models can be effective in managing conditions with significant clinical and economic burden, as well as more complex cases of patients with multiple morbidities (Dueñas et al. 2016; Rohwer et al. 2023).

In this perspective, we highlight the importance of the **Innovative Disease Management (IDM) approach.** To this aim, we first showcase examples of chronic disease that would benefit from IDM approaches. Then, we defined the main pillars of the IDM strategy at a multiscale, analyzing the impact of innovative solutions on clinical and economic outcomes.

### Examples of chronic Diseases as target of innovative Disease Management approach

#### Heart Failure

Heart failure is one of the most significant health problems in Italy, with an estimated prevalence of 1.4% of the population in 2009 and an expenditure of 2.4% of the national healthcare budget (Di Somma et al. 2020). Furthermore, 7% of patients die during their first hospitalization, and within one year of discharge, 24% of patients die and 59% are re-hospitalized. These outcomes are driven by systemic and wide-ranging causes, including malfunctions of the clinical and operational pathway (e.g., delays in referral and late diagnosis; lack of management of long-term disease; poor follow-up protocols and ongoing patient support, etc.) (Di Somma et al. 2020).

#### Chronic pain

In Italy, in 2006 the prevalence of chronic pain was estimated as high as 26%, and the burden of the disease is expected to grow with the increase of the average age of the population (Varrassi et al., 2008; Allegri et al. 2015; Millis et al., 2019). In 2015, the impact of chronic pain direct costs on the Italian public health expenditure resulted equal to 9.6% and indirect costs related to sickness leaves and retirements were also found to be significant (Allegri et al. 2015; Millis et al., 2019). Chronic pain is also associated with the highest rates of years lived with disability in all high-income countries (Millis et al., 2019).

#### Stroke

In 2011, the annual incidence rates of stroke in Italy ranged from 175/100,000 to 360/100,000 in men and from 130/100,000 to 273/100,000 in women (Sacco et al. 2011), and are expected to dramatically increase in the coming years (Howard and Goff 2012). Thirty-day case-fatality rates for all strokes ranged from 18.1 to 33% while one-year case-fatality rates ranged from 37.9 to 40.2% (Howard and Goff 2012). Data from selected Italian registers on stroke incidence and case-fatality indicate the great burden of the disease on the Italian healthcare system (Sacco et al. 2011). Beyond vital prognosis, which affects the years of life lived, stroke patients have an increased risk of poor outcome within the first year of the event that affects health related quality of life (Sacco et al. 2011; Howard and Goff 2012; Grefkes and Fink 2020).

#### Chronic Kidney Disease

According to recent data, in Italy to 3.5 million people have chronic kidney disease (CKD), which corresponds to 6.3% of the total population. Late stages of the disease bring patients to dialysis or kidney transplantation (Mancini and Santoro 2019). The Italian Dialysis Registry reports about 4500 patients treated with peritoneal dialysis (PD) and 42000 with hemodialysis (HD) corresponding to a total of 46500 patients (Mancini and Santoro 2019). The negative impact of the COVID-19 pandemic was demonstrated by numerous studies aiming at addressing the challenges faced by patients with kidney disease and their caregiver (Mahalingasivam et al. 2022).

### Innovative approaches for Disease management

The digitization of technologies, processes and services constitutes one of the main innovations in the healthcare environment (Beckmann et al. 2021). The digital revolution is related to the adoption of innovative technological solutions for data connectivity, data control and application to sectors of strategic interest and with a significant socio-economic impact (Beckmann et al. 2021).This revolution is now also being spurred by emerging data-driven technologies, artificial intelligence, big data, automation and robotics, and might lead to the development of new materials and integrated devices, supporting the exponential increase in interconnection and data processing capabilities (Cresswell et al. 2020; Blom et al. 2021).

#### Remote monitoring of outcomes

Devices for remote patient monitoring are central to helping clinicians collect patient data and monitor disease (Ruiz Díaz et al. 2020). These technologies are plentiful, ranging from wearable devices and implantable devices equipped with risk stratification algorithm (Güemes Gonzalez et al. 2020), to bluetooth blood pressure cuffs and glucose monitors. The remote patient monitoring devices usually allow clinicians to view and analyze patient-generated health data and flag irregularities. These devices have been part of the digital health conversation for several years, but now as healthcare is increasingly virtual, they may find a more permanent place in patient care (Güemes Gonzalez et al. 2020; Ganzer and Sharma 2019).

Diagnostic and therapeutic systems are able to interface at various levels with biological systems, generating data flows of clinical/physiological interest (Güemes Gonzalez et al. 2020; Ganzer and Sharma 2019; Maiolo et al. 2021; Dagliati et al. 2018). The efficient use of the currently developed flows, from the technology experimentation phase to the clinical phase, makes necessary demands to address the following challenges:

i) **the development of innovative biomedical applications and devices**, based on advanced materials capable of satisfying multiple requirements, from biocompatibility to the efficiency of application to sustainability/circular economy requisites (Torricelli et al. 2021; Bettinger et al. 2020). In this respect, bioelectronic medicine provides a new means of addressing disease via the electrical stimulation of tissues with consolidated therapeutic approach in cardiovascular disease, and with exceptional promise in the treatment of neurological and neuropsychiatric disorders (Rivnay et al. 2017). Thus, bioelectronic devices with wearable, implantable or highly integrated forms are serving as the technical basis for the state-of-art personalized medicine (Vitale and Litt 2018). They enable continuous health monitoring and on-demand medical therapy at the point of care. However, significant challenges remain to bring a variety of bioelectronic technologies to patients at scale and to provide reliable results long term. As an example, the mechanical mismatch between bioelectronic neural interfaces and brain tissue induces inflammatory gliotic reaction (Maiolo et al. 2021) that still limits the biocompatibility over time of these devices (Redolfi Riva and Micera 2021; Lacour et al. 2016; Wurth et al. 2017). The latter issue is relevant to achieve stable and efficient interfaces between electronics/cells, tissue, organs and the human body, need to ensure the reliability of data over time (Maiolo et al. 2021), which is a prerequisite for chronic disease management. With respect to bioelectronic sensors, they are ideally suitable for providing an electronic response for direct digital data communication and large source of data from the patient (Torricelli et al. 2021), but require high fidelity in their fabrication process, data reproduction and limit of detection need still to be addressed for reliable clinical testing (Macchia et al. 2022; Thompson et al. 2002; Parkula et al. 2020; Arnaout et al. 2021).

ii) **the integration between biomedical devices, advanced platforms for data elaboration and clinical/medical protocols for intervention.** Besides issues that may affect patients’ data sampling, challenges are also represented by the transmission, storage and analysis of data in a closed-loop architecture (Vitale and Litt 2018), and by the elaboration of data in terms of clinical decision and patients’ management. Growing evidence is demonstrating the benefit of artificial intelligence and big-data technologies applied to systems and protocols for healthcare management (Topol 2019) integrated sensors and point-of-care diagnostic (Ballard et al. 2020), and for prevention diagnosis and treatment of a series of health conditions and pathologies (Davenport and Kalakota 2019; Topol 2019; Wang et al. 2023; Lee et al. 2018). Machine learning approaches for intelligent biosensing and data recognition are used to analyze data appropriately, draw the right conclusions from the data, and recognize if the data has been misinterpreted. With respect to point-of-care diagnostic, digital platform capable of artificial intelligence-based binary classification at the limit of identification of a single biomarker have been recently reported (Macchia et al. 2022). Thus, Artificial Intelligence (AI) might help overcome challenges related to data collection, data consistency, monitoring accuracy and reliability and not only to data analyses. AI approaches can also perform complex tasks which may be useful for therapeutic intervention based on bioelectronic implants, such as in closed-loop neural interfaces (Massey et al. 2019; Datta-Chaudhuri 2021). Application of data-driven technologies to growing number advanced devices (high-resolution medical imaging, biosensors with continuous output of physiological parameters, genome sequencing) as well as to electronic medical records could bring unprecedented innovations in diagnostic and clinical intervention (Topol EJ, 2016; Debnath et al. 2020). A further milestone will be achieved leveraging on these approaches to create risk stratification models and enhance patient management based on data-driven decision.

#### Patient-centric care models

**Fig. 1:**
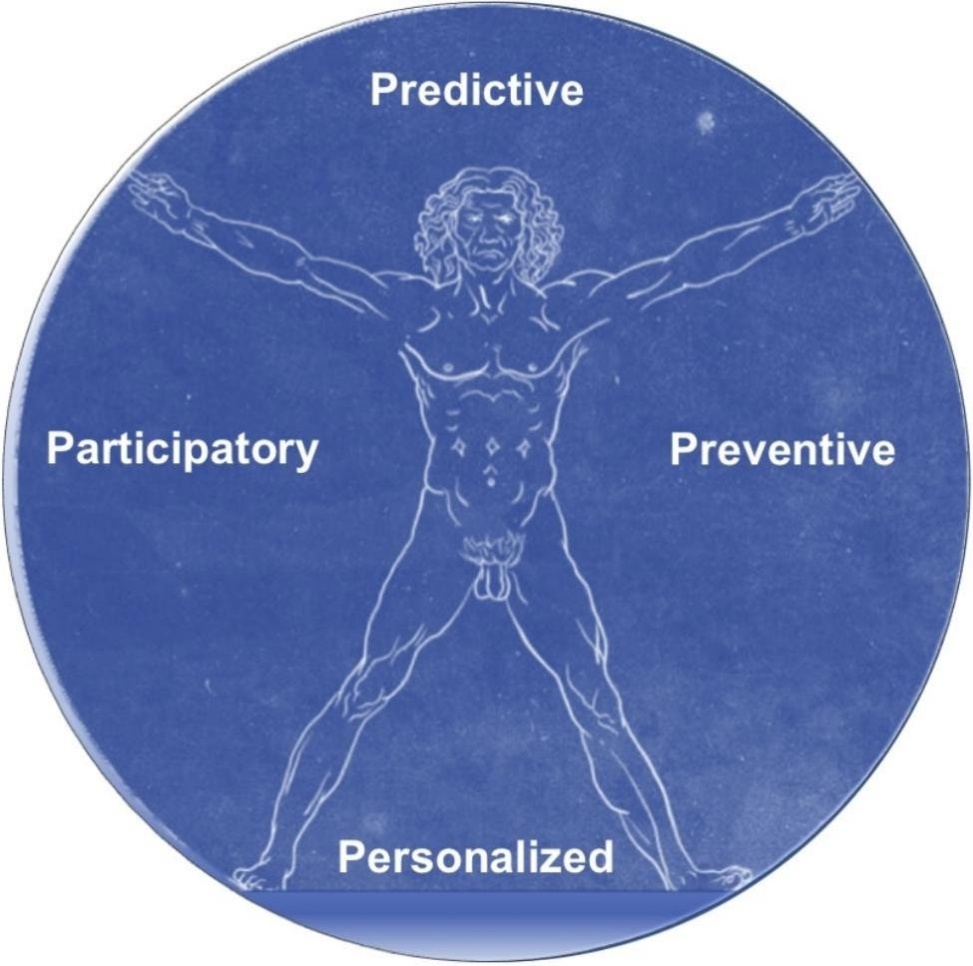
Scheme representing the 4 Ps of the Personalized Medicine Approach

Another crucial aspect of this shift in paradigm is the involvement of patients in the prediction, prevention, and personalization of their care. Ensuring patients will move from a passive receiver of care role to an active and conscious contributor to their wellbeing, can improve timely access to the detection of adverse medical and behavioral events and stimulate the achievement of the P4s of Precision Medicine: predictive, preventive, personalized, and participatory (Bloom et al., 2021) (Fig. 1).

The rising importance given to patient involvement is also facilitated thanks to the recent innovations in Big Data technology and medical informatics. Big Data technologies are providing new powerful instruments to gather and jointly analyze large volumes of heterogeneous data collected for different purposes, including clinical care, administration, and research. However, the collection of data from simultaneously operating system demands an infrastructure that can be viewed as an integrated set of services supporting all of the simultaneous needs emerged by the elaborated data set. In fact, without such enabling infrastructure, it would not be possible to collect, store and process large amounts of patient feedbacks as well as to create predictive models and related services, based on the collected data (Friedman 2022).

#### Enabling technologies for Disease management

Emerging information technologies in the health sector can be applied to many dimensions of disease management, including health data collection and aggregation, patient remote monitoring and continuous assistance, and diagnostic and therapeutic protocol improvements. Consequently, digitalization in the healthcare sector will have an increasingly pervasive role, affecting the management of pathology with a high impact on aspects of people’s lives (Dale et al. 2014; Frank et al. 2019). All stakeholders involved benefit from the innovations in the sector, as patients, healthcare operators, and the wider health management system have gains from improved safety, access and quality of care, efficiency, and cost reductions.

Bioelectronic systems for data capture, analysis and visualization aim to generate knowledge and technology to fill gaps in the current diagnostic and therapeutic protocols through an integrated strategy based on innovative patient centric approaches, looking for new ways to promote health and reducing costs (Dale et al. 2014; Frank et al. 2019). The proper design of interactive dashboard-based tools may enable precise medicine decision-making and case-based reasoning. Leveraging these tools can allow a proper organization and visualization of data collected, which might also highlight clinical patterns not previously considered. Formal models of clinical guidelines and care pathways can be very effective tools to compare the analytics results with expected behaviors. This may enable the effective revision of routinely collected data, the generation of new insights about patients’ outcomes, and new interpretation of clinical patterns.

Considering these opportunities, one of the most relevant factors impacting healthcare digitization concerns the multi-level management of data, from the data capture to the institutional management (electronic health cards, genomic data, etc.), of fundamental importance for the definition of protocols and policies. Numerous initiatives at the European level have already identified the main characteristics of health data management systems, which essentially concern the quality of health data, the characteristics of interoperability, the support for infrastructures and the management systems related to regulations.

#### Integrated care and interdisciplinary approach

**Fig. 2:**
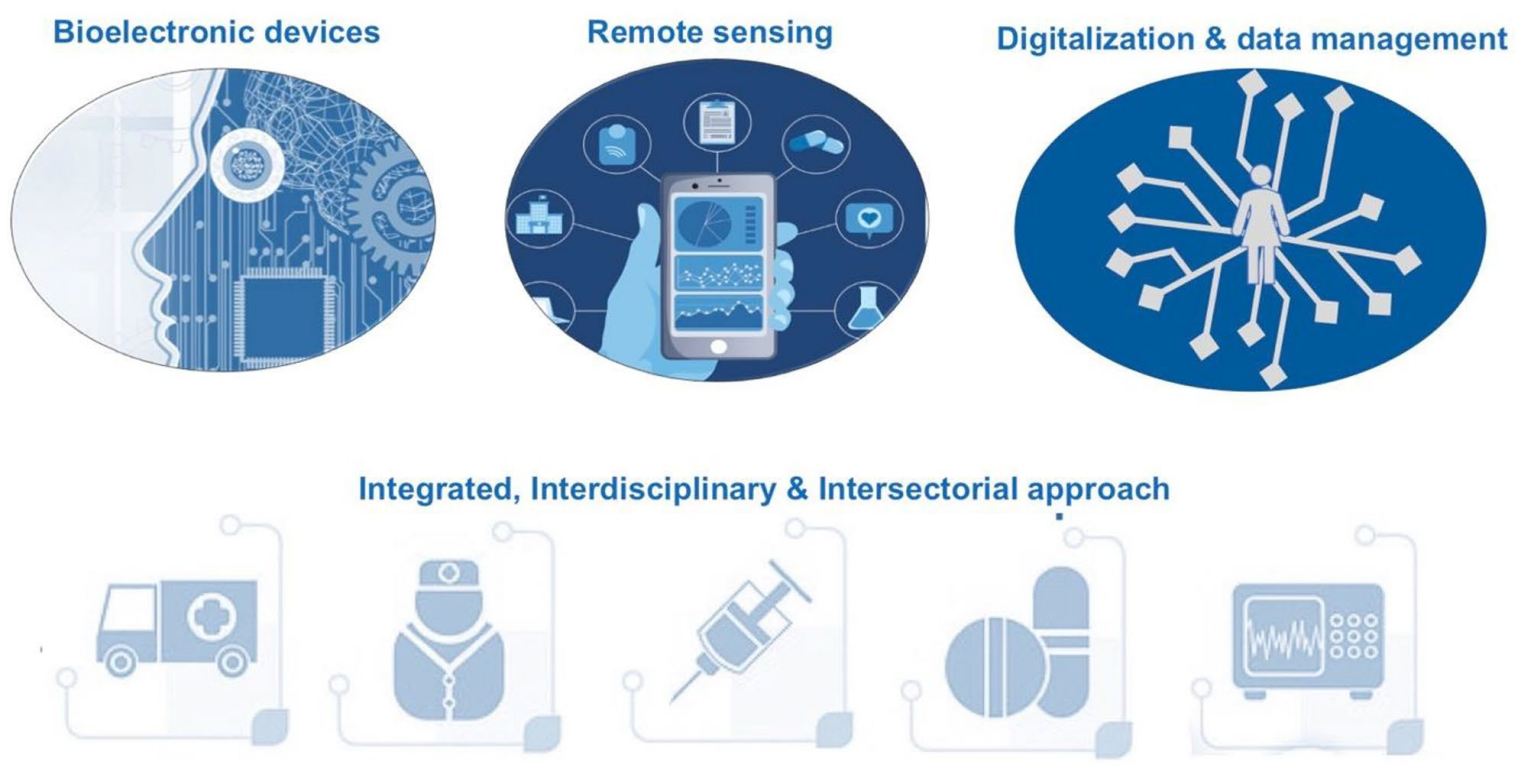
Scheme of the proposed pillars of Innovative Disease Management approach

Innovative solutions for disease management must therefore cover a broad spectrum of technologies and skills, advanced materials (Maiolo et al. 2021; Kuipers et al. 2019) integrated in enabling technologies for the development of health devices (Dale et al. 2014; Frank et al. 2019; Kuipers et al. 2019), overall engineering and development of interconnected devices and developing data analysis platform for clinical-medical support (Fig. 2). The design and realization of integrated, patient-centric diagnostic and therapeutic tools requires the synergy of a large series of technologies and skills. Multidisciplinary collaborations are necessary, considering the complexity and the variety aspects to consider. As an example, in the Italian healthcare system, there are successful models of integrated care for heart failure, in Bergamo and Trieste, where care is multidisciplinary and includes home visits and telemedicine appointments to support transition from hospital to community care. Another model developed by The Italian Association for Heart Failure (Associazione Italiana Scompensati Cardiaci, AISC) based on self-care education and patient empowerment has resulted in improved outcomes. All of these models could benefit from wider roll-out to improve the response to the challenge of heart failure (Dale et al. 2014).

### The impact of optimizing Disease management

#### Impact on clinical outcomes

The impact of optimizing infrastructural aspects of patient care management on clinical effectiveness, safety, and quality of life is an emerging topic in the existing literature. The interventions observed are mostly patient-centered approaches, lean management processes and tools, platforms, and apps for patient data monitoring, reporting and analysis. For instance, one study demonstrated that effective coordination of care and multidisciplinary rounds can improve patient-centric measures of care quality and safety (Frank et al. 2019; Kuipers et al. 2019; Hudson et al. 2016). Other findings show that patient-centric care and co-creation of care are positively associated with patient satisfaction, physical and social well-being, even more in case of patients with multi-morbidity, therefore with higher complexity and heterogeneity in management (Kuiperset al. 2019).

Studies in the US have examined the effect of focusing on patient experience and quality of care (Everink et al., 2018). Positive associations were found between patient experience and overall technical quality and safety. For patients with acute myocardial infarction, several studies have shown an association between patient-centric care and clinical outcomes, including mortality. One study found that applying better patient-centric care during hospitalization for infarction was associated with a decreased risk of death one year after discharge. Other clinical benefits that have been found to be associated with a better patient experience and patient-centric care include decreased rates of healthcare acquired infections, improved delivery of preventive care services, reduced length of stay, improved adherence to treatment regiments and improved functional status. Better communication with physicians, clean and quiet hospital environment and the responsiveness of hospital staff were significantly associated with a decreased likelihood of pressure ulcers and post-operative infections. In addition, stronger patient engagement and education about their disease, has been found to reduce the risk of experiencing an adverse event. Patient centric care has also been associated with a reduction in the number of diagnostic tests and other referrals, better adherence to treatment regiments, greater patient satisfaction, and greater patient enablement.

Integrated care models also have a significant impact on clinical outcomes. One research found that for patients with different chronic conditions, the risk of hospitalization is reduced by 19% on average. The integration of services for disease management can also reduce the risk of readmissions, visits, and length of stay (Stephenson et al. 2019). For stroke a report was developed by the Organization for Economic Co-operation and Development (OECD) to assess the impact of integrated care on clinical outcomes at international level (Barrenho et al. 2022). The study concludes that evidence on the impact is mixed, with improved patient satisfaction but modest effects on outcomes, care utilization, mortality or spending, while results from mostly local-level experiences suggest some effects like better access, improved satisfaction for patients and workforce, reduced hospital utilization such as (re)admissions rates, emergency visits, and delayed admissions to institutional care, and improved quality of life and preventive care. A systematic review of randomized trials on disease management for heart failure (McAlister et al., 2001) found that specialized follow-up by a multidisciplinary team leads to a substantial reduction in the risk of hospitalization. For chronic pain, a study on lower back pain (Loisel et al. 1997) for instance, observed the impact of providing usual care versus improved management and intervention on the functional status, pain, and duration of absence from work. The full intervention group was found to return to regular work 2.41 times faster than the usual care intervention group. Pain and disability scales demonstrated either a statistically significant reduction or a trend toward reduction in the intervention groups, compared with the trend in the usual care. In the field of neuropathy, optimizing the clinical pathway, targeting an earlier detection is associated to clinical benefits for patients as reductions in the exposure to nephropathic drugs, changes in diet and lifestyle, slower kidney failure progression and improvements in education and awareness on the disease (Hudson et al. 2016). Moreover, the optimization of the clinical pathway, can facilitate the creation of a network between nephrologists and other specialists (cardiologists, diabetologists, GPs, etc.) in placing the patient at the center of the care pathway. For patients who will reach the end stage of the disease and need to start a dialysis treatment, they can be well prepared for dialysis success (less complications) by early preparation of care access, moreover being also able to choose the applicable treatment that better fits their lifestyle. This last opportunity can help patients to reduce the significant psychological distress associated to dialysis, as they are presented with the possibility of finding the treatment that has less impact on their quality of life (Hudson et al. 2016).

#### Impact on economic outcomes

The cost-effectiveness of optimized patient pathway and patient-centric care have been analyzed in several studies. Within economic evaluations, integrated care pathways often result as cost-effective interventions, meaning that the additional health benefits gained from optimized patient management can compensate the additional investments. One publication investigated the cost-effectiveness of an integrated care pathway for older patients with complex health problems (Everink et al. 2018). The results of this study indicate that the integrated care pathway is a cost-effective intervention, with significantly lower average societal costs for patients in the care pathway cohort (€50,791) versus patients in the care as usual cohort (€62,170). Another study on cost-effectiveness showed that patient centric care entails lower costs and improved effectiveness as compared to usual care, for a 2-year time and a 5-year perspective (Pirhonen et al. 2020).

Many articles investigate the impact of innovative solutions for patient management on cost drivers such as hospital staff or patient length of stay. Patient centered approaches are generally associated with shorter average length of stay, statistically significant lower cost per case, shift in emphasis from the use of higher-cost staff to lower-cost staff and higher-than-average overall patient satisfaction score. One study found a decrease in mean length of stay decreased from 4 hospital days to 3.6 hospital days, representing a 10% relative reduction (Kuipers et al. 2019).

In addition to patient pathway or lean healthcare management, data on the economic impact of several digital solutions is also available. For instance, a decisive role for potential savings has been identified in the “Health Care Internet of Things”, which regards the use of sensors, smartphone applications and remote monitoring for the continuous collection of clinical information, data in cloud to allow clinicians to access information of patients treated at home, in their clinic, or elsewhere, allowing their management across different specialists and geographical areas. The main application of the Internet of Things in hospital settings is within the flow and traceability of patients and the usage of healthcare technologies. In Italy it was estimated that a 1% increase in the efficiency of these processes can result in savings of €1 billion each year.

National studies have shown the benefits of telemedicine, in terms of both feasibility and patient adherence, in the stabilization of heart failure symptoms and the reduction of hospital readmissions and associated healthcare costs. At the beginning of the COVID-19 pandemic, a seven-week pilot telemedicine service, which included 24 h/7days phone access to healthcare professionals with chat and videoconferencing options, reduced heart failure hospitalizations and deaths compared with the same period in 2019. This highlights the value of telemedicine as an important tool for the management of heart failure and its potential in supporting more developed integration of care.

It is estimated that in the EU €99 million can be saved with the implementation of mobile health (mHealth), i.e. mobile technologies and solutions that enable healthcare delivery solutions. In addition to savings, through these solutions patients are expected to improve their lifestyle, how they manage their medical condition, and their involvement in managing their own disease. In terms of indirect economic impact, mHealth could enable 11.2 million chronic patients and 6.9 million patients at risk of developing chronic diseases to extend their professional lives and improve their productivity (for example, by increasing their quality of life or reducing time for transportation). The latter factor was estimated to add €93 billion to the EU GDP in 2017.

### Proposed Framework for innovative approaches for Disease management

#### Framework Design & Clinical Integration

Considering the models for disease management presented and their expected clinical and economic impact, we propose a continuum of innovative and integrated services to cover the entire patient pathway. Integrated services for disease management can include combinations of analyses to identify needs, strategies for improvement of patient and caregiver outcomes, and design of new analytic and prediction models to obtain patient centric care.

Examples of analysis of the patient pathway and unmet needs are literature and guidelines assessments, advisor boards, gap analyses and project planning. The identification of methods to improve outcomes are, for instance, risk stratification and risk-based clinical intervention models, as well as optimal patient pathway definition and referral network optimization. In the phase of implementation, it is necessary to provide tools to support clinical decisions, to optimize follow-up management and to generate insights from elaboration of real-world data.

#### Impact on patient outcomes and staff impact

According to literature innovative models for disease management as well as their integration can have a significant impact on clinical outcomes and resource use, which in turn can generate economic savings for National Healthcare Systems. The added value from designing strategies that combine both innovation and integration has also been addressed in the literature. A publication on the role of digitalization on integrated care (Shah et al. 2022) argues that the incorporation of technology in healthcare is essential to drive an integrated model of care - one which is holistic, patient-centred, preventative and shows clear communication between different specialties, providers, and levels of care. The researchers conclude that advances in technology and its application to healthcare are leading to explosive growth in virtual consultations, remote monitoring mobile health, digital therapeutics, and artificial intelligence/machine learning. Not only have their adoption led to more integrated care systems, but also more cost effective, efficient, and higher quality models at scale. An example is a recent data-driven and device-based algorithm applied to the care pathway of patients with heart failure (Ahmed et al. 2022). The algorithm integrates data from different sources to stratify patients based on the risk of hospitalization, and triggers remote intervention when cases of ‘high’ risk are identified. This combination of innovative and integrated services was associated with a reduction in hospitalizations equal to 58% and minimal staffing time.

#### Data Quality & Use

The main challenge with implementing innovative and integrated solutions for disease management is tied to the current state of the management of patients and the healthcare data landscape, i.e. whether it is fit-for-purpose or usable in the present state to power the proposed IDM framework. For this reason, before proposing a model of integrated services for disease management, it is crucial to analyze the current state of the care giver system and to propose solutions that are tailored to their existing needs. In some cases, the integration of services and solutions for disease management can have the primary aim of achieving a data landscape that can allow precision medicine.

## Conclusions

On the basis of the evidences collected, we propose a model that can guide changes in research and innovation of the health system today. Such model is based on the main pillars of innovative disease management, such as precise medicine and patient-centric approaches, as well as lean, optimized, and therapy-specific patient pathways.

Disease management has the mission of creating innovation for the entire digitalization chain in the medical / health sector, from wellness, to diagnostics, to high-impact pathologies. One of the major objectives to be achieved is to improve the condition of patients and the health of the person, reducing the costs associated with therapy and any complications through technologies and innovative approaches that allow rapid and effective diagnosis and treatment.

In this perspective, we envision a platform where people’s care can follow personalized, inclusive, continuous and shared paths. The strategy we propose to achieve this goal should be based on an open innovation, one-health approach, capable of generating research, innovation and value proposition in the fundamental aspects of managing medical / clinical platforms. Innovative solutions for disease management include the use of technologies or services that can enable patient-centric care and optimization of patient pathways. This evaluation revealed that adopting these strategies is beneficial not only under a clinical perspective (safety, effectiveness, and quality) but also under an economic perspective (direct and indirect savings).

At the same time, we believe there is a lack of infrastructural reference networks in the field of applications of advanced materials for health, in the development of devices, in the generation, transmission and processing of data of health and diagnostic interest. The specific competences of the infrastructure are needed to integrate innovative biomedical and bioelectronic devices solutions, (including lab-on-a-chip systems and point-of-care tests,) with advanced platforms for information processing based on artificial intelligence and big data.

The needed private-public-partnership must be designed to favor interaction with external research entities and with the production sector, supporting the processes of knowledge valorization and technological transfer through cooperation agreements with start-ups and companies in the area and through actions at national level for digitalization in the health sector. Within this framework, Medtronic Study & Scientific Solutions (S&SS) is collaborating with the Italian National Research Council (CNR), other internal Medtronic groups and external partners to develop integrated solutions for the optimization of chronic disease management.

In the medium to long term, the platform aims to create innovation and skills in the digitalization paradigm based on today’s heterogeneous technologies and approaches for diagnostics and advanced therapy. From information on basic biological mechanisms, to the development of advanced biomedical materials and devices, to the analysis of complex health data and the realization of predictive models for risk-segmentation, the platform will generate life-transforming technologies capable of radically changing the approach to health.

## Data Availability

Not applicable.
